# Plasmid Replicon Typing of Antibiotic-Resistant *Escherichia coli* From Clams and Marine Sediments

**DOI:** 10.3389/fmicb.2020.01101

**Published:** 2020-05-27

**Authors:** Barbara Citterio, Francesca Andreoni, Serena Simoni, Elisa Carloni, Mauro Magnani, Gianmarco Mangiaterra, Nicholas Cedraro, Francesca Biavasco, Carla Vignaroli

**Affiliations:** ^1^Department of Biomolecular Sciences, Biotechnology Section, University of Urbino “Carlo Bo”, Urbino, Italy; ^2^Department of Life and Environmental Sciences, Polytechnic University of Marche, Ancona, Italy

**Keywords:** PBRT kit, plasmid, Inc group, replicon, antimicrobial resistance, *Escherichia coli*

## Abstract

Unlike human isolates, environmental *Escherichia coli* isolates have not been thoroughly investigated for the diversity and transferability of antibiotic-resistant plasmids. In this study, antibiotic-resistant strains from marine sediment (*n* = 50) and clams (*n* = 53) were analyzed (i) for their plasmid content using a PCR-based plasmid replicon typing (PBRT) kit and (ii) for the transferability of plasmid-associated antibiotic resistance (AR) traits by mating experiments. Fifteen of the thirty replicons targeted by the PBRT kit were detected in the isolates; 8/15 were identified in both sediment and clam isolates, although at different frequencies. The most frequent replicons in sediment (74%) and in clam strains (66%) alike, were FIA, FIB, or FII, which are associated with the IncF group, followed by the I1α replicon, which was more frequent in clam (24.5%) than in sediment (10%) strains. More than 50% of the strains contained multiple replicons; although 15 were untypable, S1-PFGE analysis demonstrated that 14/15 carried no plasmids. All cryptic strains were successfully typed and were positive for IncF or IncI replicons. Antibiotic-resistant strains accounted for 63% of all isolates and were significantly (*p* < 0.05) more frequent in phylogroup A. Most (35%) multidrug-resistant (MDR) strains belonged to phylogroup A, too. Although 25/26 MDR strains were positive for IncF plasmids (the exception being a clam strain), the FII-FIB rep combination was predominant (63%) among the sediment isolates, whereas most clam isolates (40%) carried the FII replicon alone. In mating experiments, selected MDR strains carrying FIB, FII, and I1α replicons, used as the donors, transferred multiple ARs together with the IncF or IncI plasmids at high frequency. Since IncI plasmids are common in *E. coli* and *Salmonella enterica* isolates from poultry, our findings suggest an animal origin to the *E. coli* clam strains carrying IncI plasmids. They also suggest a role for IncI plasmids in the spread of ARs among environmental *Enterobacteriaceae* and, through the food chain, to human isolates. In conclusion, the PBRT kit proved to be a useful tool to identify plasmids carrying antibiotic-resistant genes and to shed light on the factors underpinning their diffusion.

## Introduction

Antimicrobial resistance – a major and topical clinical issue – has also become an environmental concern owing to the growing spread and ubiquity of resistance genes and bacteria (Bengtsson-Palme et al., 2018). The human and animal microbiota appears to be the primary reactor where antibiotic resistance develops, due to exposure to the selective pressure of antibiotics administered for infection treatment or prophylaxis (Baquero et al., 2008; Rolain, 2013). Moreover, the gut may be a reservoir of resistance genes, where horizontal genetic transfer (HGT) among different microbial species may easily occur (Rolain, 2013). Human and animal antibiotic-resistant (AR) bacteria released into wastewater find their way to the soil and to water environments; in particular, water is a favorable habitat for interactions and gene exchanges among micro-organisms, the dissemination of resistance genes or bacteria, and the transmission of waterborne infectious disease (Finley et al., 2013; Jang et al., 2017). *Enterobacteriaceae* are found in a wide range of environments. The presence of *Escherichia coli* – a common bacterium of the human and animal intestinal microbiota – in natural water bodies has long been interpreted as indicating fecal contamination. More recently, *E. coli* has been demonstrated to be highly adaptable and to be able to survive and replicate outside the host, in water, soil, sediment and vegetables (Delaquis et al., 2007; Bergholz et al., 2011; Frank et al., 2011; Jang et al., 2017). Commensal as well as pathogenic *E. coli* strains resistant to several antibiotics have been recovered in the marine environment (Vignaroli et al., 2013, 2016; Charnock et al., 2014; Drali et al., 2018). Antibiotic resistance in *E. coli* species is of particular concern because of the growing prevalence of multidrug-resistant (MDR) strains involved in human and animal infections. Moreover, the incidence of AR commensal *E. coli* isolates in healthy humans is increasing worldwide (Broaders et al., 2013; Rolain, 2013), contributing to the emergence and spread of AR pathogens. AR clinical isolates of *E. coli* are also frequently related to those from animals, suggesting that both the food chain and food animals may be a source of MDR strains (Rolain, 2013; Lazarus et al., 2015; Berg et al., 2017).

Besides antibiotic exposure and HGT events, the acquisition of resistance genes by *E. coli* isolates is also affected by their genetic background. Some phylogenetic groups (i.e., A and D) are more prone to develop antibiotic resistances, and strains belonging to the same phylogroup and sequence type (ST) often share the same antibiotic resistance profile (Tenaillon et al., 2010). *E. coli* population structure can provide useful information on strain origin, since different phylogroups predominate in distinct ecological niches (Tenaillon et al., 2010). The acquisition of plasmids, which enhance resistance gene dissemination, is believed to play a key role in the growing prevalence of MDR *E. coli* strains (Mathers et al., 2015). In *Enterobacteriaceae*, some plasmids associated with specific resistance determinants are predominant in specific geographic areas; they are able to replicate in a wide host range (e.g., IncA/C and IncL/M plasmids) and their dissemination relies on antimicrobial selective pressure (Rozwandowicz et al., 2018). Other plasmids are only found in closely related hosts and are maintained by the bacterial cell because they encode virulence factors enhancing bacterial adaption and fitness (Carattoli, 2009).

This study was undertaken to determine: (i) the distribution and prevalence of major plasmid replicons in *E. coli* isolated from clams and marine sediment using the PCR-based replicon typing (PBRT) kit and (ii) the involvement of specific plasmids in the conjugal transfer of antibiotic resistance from environmental *E. coli*.

## Materials and Methods

### Bacterial Strains

A total of 103 *E. coli* isolates (53 from clams and 50 from marine sediments) were used in the study. The 53 clam strains were selected from a collection of 141 strains isolated from Venus clams collected in Italy in the middle Adriatic Sea, which had previously been characterized for antibiotic resistance phenotype and phylogroup (Vignaroli et al., 2016). The 50 sediment strains were isolated from samples collected at three coastal sites (SE, PN, and API) at a depth of 4–12 m (from latitude 43°45.300′N, longitude 13°12.630′E, to latitude 43°39.0′N, longitude 13°22.0′E) near the areas where the clams had been harvested.

To detach bacteria from sediment, aliquots (5 g) of each sample were suspended in 20 mL sterile seawater, vortexed and sonicated (3 times, 1 min per cycle) as described previously (Luna et al., 2010; Vignaroli et al., 2013). The resuspensions were filtered by the membrane filter technique and *E. coli* strains were isolated in the selective medium mFC agar (BBL, Becton Dickinson & Co., Sparks, MD, United States) (Vignaroli et al., 2013).

### Strain Identification and Typing

Presumptive *E. coli* colonies from sediment samples were identified by the molecular approach based on PCR amplification of the species-specific *uidA* gene (McDaniels et al., 1996). The PCR methods developed by Clermont et al. (2011, 2013) allowed each *E. coli* isolate to be assigned to a phylogenetic group or cryptic clade.

### Antimicrobial Susceptibility and PCR Detection of Class 1 Integrons and Antibiotic Resistance Genes

Strain susceptibility to ampicillin (10 μg), cefotaxime (30 μg), gentamicin (10 μg), ciprofloxacin (5 μg), tetracycline (30 μg), chloramphenicol (30 μg), nalidixic acid (30 μg), trimethoprim/sulfamethoxazole (1.25/23.75 μg) and streptomycin (10 μg) was assessed by the disk diffusion method according to CLSI recommendation (Clinical and Laboratory Standards Institute (CLSI), 2017). Strains resistant to β-lactams were analyzed for extended spectrum β-lactamase (ESBL) production by screening and confirmatory tests (Clinical and Laboratory Standards Institute (CLSI), 2017). In brief, a standard disk diffusion test was performed in which the β-lactam antibiotic disks of both cefotaxime and ceftazidime, alone and in combination with clavulanate were used. A ≥5-mm increase in a zone diameter for either antimicrobial agent tested in combination with clavulanate vs. the zone diameter of the agent when tested alone indicated the ESBL production by the strain (Clinical and Laboratory Standards Institute (CLSI), 2017).

*E. coli* ATCC 25922 was the reference strain in all antimicrobial susceptibility assays. Resistant strains were screened by PCR for the following determinants: *bla*_*TEM*_, *bla*_*SHV*_, and *bla*_*CTX–M*_ (ESBL-encoding genes) for β-lactam resistance; *tet*(A) for tetracycline resistance; *dfrA1* for trimethoprim/sulfamethoxazole resistance; and *strA*, *strB*, *aadA*, and *ant*(3″) for aminoglycoside resistance. Primers and PCR conditions were as reported previously (Vignaroli et al., 2012, 2013).

MDR strains were analyzed by PCR for the *intI1* integrase gene and the variable region of the class 1 integron using primers and PCR conditions described previously (Vignaroli et al., 2012). The resistance genes linked to class 1 integron were characterized by sequencing (GATC Biotech Cologne, Germany) the cassette amplicons and by nucleotide sequence analysis using the Basic Local Alignment Search Tool (BLAST)^1^.

### Plasmid Typing

The PBRT 2.0 kit (Diatheva, Fano, Italy) which has been used for plasmid identification and to type the major resistance plasmids found in *Enterobacteriaceae* (Carloni et al., 2017), was applied in our study. The amplicons recognized by the PBRT kit were analyzed by capillary electrophoresis with an AATI Fragment Analyzer (Agilent, Santa Clara, CA, United States).

Where necessary, amplicons were purified using MinElute PCR purification kit (Qiagen, Hilden, Germany) and directly sequenced by the Sanger method using BigDye Terminator v. 1.1 Cycle Sequencing Kit (Thermo Fisher Scientific, Vilnius, Lithuania) on the ABI PRISM 310 Genetic Analyzer (Applied Biosystems, Thermo Fisher Scientific, Waltham, MA, United States). The consensus sequences obtained were submitted to the pMLST database for allele variant identification^2^.

The *stx1*, *stx2*, and *eae* genes of shiga toxin-producing *E. coli* (STEC) strains were identified using STEC FLUO detection kit (Diatheva, Fano, Italy) according to the manufacturer’s instructions; the strains that were positive for *stx* and/or *eae* genes underwent O-serogroup identification (O157, O111, O26, O103, and O145) using the STEC Serotypes FLUO kit (Diatheva).

### S1-PFGE

S1-nuclease pulsed field gel electrophoresis (S1-PFGE) was performed to determine plasmid size and compare the plasmid profile of strains. Total DNA embedded in 0.8% agarose plugs was incubated for 30 min at 25°C with 100 U *Aspergillus oryzae* S1 nuclease (Takara Bio Inc., Shiga, Japan). The plugs were loaded on a 1% agarose gel using 0.5X TBE running buffer. Electrophoresis was performed in a CHEF-mapper system (Bio-Rad Laboratories, Inc., CA, United States) with the pulse time increasing from 1 to 25 s for 17 h at 14°C and 200 V (6 V/cm). The Low Range PFG Marker (0.1–200 kb) and Lambda Ladder PFG Marker (50–1,000 kb) from New England Biolabs (Ipswich, MA, United States) were used as molecular size markers.

### Conjugation Experiments

Conjugal transfer of tetracycline and β-lactam resistance was performed by filter mating using the protocol previously described (Vignaroli et al., 2011). The *E. coli* 1816 (a mutant of *E. coli* C600, lactose-non-fermenting, resistant to nalidixic acid and rifampicin) was used as the recipient strain. Transconjugants were selected on Brain Heart Infusion agar (BHIA) (Oxoid, Basingstoke, United Kingdom) supplemented with tetracycline (20 μg/mL), rifampicin (50 μg/mL), and nalidixic acid (50 μg/mL). Transfer frequency was expressed as number of transconjugants per recipient. Transconjugants were first confirmed by three passages on MacConkey agar (Oxoid) containing all three antibiotics at the concentrations used for selection. Plasmid acquisition was assessed by comparing the S1-PFGE profiles of transconjugant and donor and confirmed using the PBRT kit.

### Hybridization Assays

The plasmid location of the *tet*(A) gene was investigated in the donors, the transconjugants and the recipient *E. coli* 1816 by hybridization assays after S1-PFGE and Southern blotting. DNA was blotted onto positively charged nylon membranes (Bio-Rad Laboratories) and hybridized with a biotin-labeled *tet*(A) probe using North2South^TM^ Chemiluminescent Hybridization and Detection Kit (Thermo Fisher Scientific, Rockford, IL, United States), according to the manufacturer instructions.

### Statistical Analysis

Differences in the prevalence of the replicon types were analyzed by the χ^2^ test. The significance of the association between a replicon type and resistance to a specific antibiotic or belonging to a specific phylogroup was analyzed by Fisher’s test. A *P*-value < 0.05 was considered significant.

## Results

A total number of 103 *E. coli* isolates, obtained from clam and sediment samples collected at roughly the same three sites (API, SE, and PN) were analyzed in this study. The 53 *E. coli* clam strains belonged to different phylogroups; most (*n* = 31) were from clams harvested next to the API site, 10 came from site SE and 12 from site PN. Most isolates belonged to phylogroup A (*n* = 21), B1 (*n* = 7) or D (*n* = 6) whereas six were cryptic (Vignaroli et al., 2016; Table 1).

**TABLE 1:** Prevalence of the replicons detected by the PBRT kit in *E. coli* isolates from clams and sediments.

Phylogroup	N. of strains	N. of strains positive for each replicon type																	
		HI1	I1α	I1γ	X1	X3	X4	M	N	FIA	FIB	FII	FIB KN	K	B/O	R	More Rep	No Rep^a^
**Clams**																		
A	21	1	3	–	4	1	–	–	1	2	3	12	–	–	–	–	7	3
B1	7	–	1	–	–	–	–	–	–	–	3	4	2	–	–	–	3	–
B2	4	–	1	–	–	–	–	–	–	2	3	3	–	–	–	–	3	–
C	2	–	1	–	–	–	–	1	–	–	1	1	–	–	–	–	1	1
D	6	–	1	–	–	–	–	–	–	1	4	4	–	–	–	–	4	1
E	5	–	1	–	–	–	–	–	1	1	3	2	–	–	–	–	2	2
F	1	–	1	–	–	–	–	–	–	–	–	–	–	–	–	–	–	–
U^b^	1	–	–	–	–	–	–	–	–	1	1	1	–	–	1	–	1	–
clade III-IV-V	6	–	4	1	–	–	–	–	–	–	2	3	–	–	–	–	4	–

Total	53	1	13	1	4	1	–	1	2	7	20	30	2	–	1	–	25	7

**Sediments**																		
A	16	3	2	–	–	–	–	–	–	–	4	9	1	–	–	–	7	4
B1	13	–	3	–	1	–	–	–	–	1	6	7	2	–	–	1	6	2
B2	10	–	–	–	–	–	1	–	1	4	5	7	–	1	–	–	7	2
C	3	–	–	–	–	–	–	–	1	–	3	3	–	–	–	–	3	–
D	4	–	–	–	–	–	–	–	–	1	2	4	–	1	–	–	2	–
E	–	–	–	–	–	–	–	–	–	–	–	–	–	–	–	–	–	–
F	2	–	–	–	–	–	–	–	–	–	2	2	–	–	–	–	2	–
U^b^	–	–	–	–	–	–	–	–	–	–	–	–	–	–	–	–	–	–
clade V	2	–	–	–	–	–	–	–	–	–	2	2	–	–	–	–	2	–

Total	50	3	5	–	1	–	1	–	2	6	24	34	3	2	–	1	29	8

*^*a*^Negative for all replicon types detected by the PBRT kit. ^*b*^Unknown, PCR-negative for phylogroup determination (Clermont et al., 2013).*

Analysis of the 50 isolates from sediment samples grown on mFC agar and identified as *E. coli* showed that most of them (*n* = 41) were from site PN and that they belonged to all phylogroups except E and F. There were only two cryptic strains, both of clade V, from site PN (Table 1).

All 103 strains were subjected to plasmid replicon typing; the results are reported in Table 1. Overall, 15 of the 30 replicons detected by PBRT kit were found in the strains; 8/15 were shared by clam and sediment strains, although at different frequencies. FIA, FIB, and FII, carried by IncF family plasmids, were the most frequent types, followed by replicon I1α, which is associated with IncI plasmids. IncF plasmids which were detected in 74% of sediment and 66% of clam strains carried single or multiple IncF replicons. In particular, the rep combination FII-FIB was significantly (*p* = 0.03) more frequent in sediment (54%) as well as in clam (28.6%) strains. More than 50% of strains contained multiple replicons, whereas 15 (8 from sediments and 7 from clams) were untypable by the PBRT kit. S1-PFGE analysis showed that 14/15 untypable strains did not contain plasmids. Interestingly, replicon I1α was more frequent among clam (24.5%) than among sediment strains (10%), but the difference was not significant. All cryptic clade strains (8) were typable by the kit and were positive only for IncF and/or IncI family replicons; 50% of these strains were positive only for FIB and FII replicons, whereas the remaining 50% carried I1α in addition to I1γ (clade III, *E. coli* ISZ 201), or FII (clade V, *E. coli* ISZ 272). In three *E. coli* clam strains (ISZ 45, ISZ 211, and ISZ 275), the PCR products of the FIA replicon were smaller (from ∼411 to ∼440 bp) than those of the control strain (462 bp). Sequencing and comparison with the pMLST database disclosed that all were FIA replicons carrying short deletions; in particular, *E. coli* ISZ 45 and *E. coli* ISZ 211 bore the FIA6 allele, whereas *E. coli* ISZ 275 carried the FIA5 allele; compared with the FIA2 allele of the control strain *E. coli* ISZ 35, the FIA6 and FIA5 alleles showed a deletion of 55 and 22 bp, respectively.

The STEC FLUO kit for the *stx1*, *stx2*, and *eae* virulence genes, to detect shiga toxin-producing *E. coli* showed that only *E. coli* PN37 from sediment was positive for gene *eae*, but that it did not belong to any of the serogroups detected by the kit.

**FIGURE 1:**
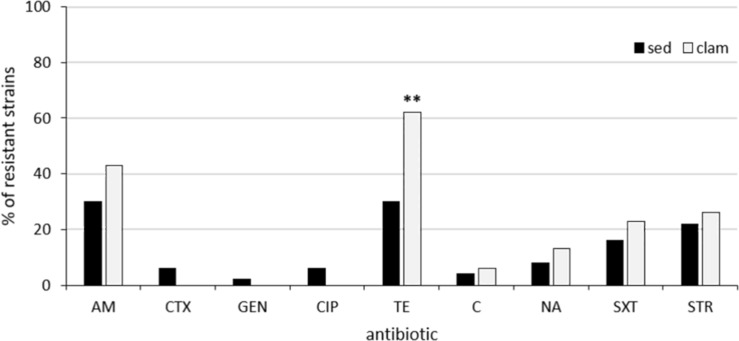
Prevalence of resistant strains among clam and sediment isolates. AM, ampicillin; CTX, cefotaxime; GEN, gentamicin; CIP, ciprofloxacin; TE, tetracycline; C, chloramphenicol; NA, nalidixic acid; SXT, trimethoprim/sulfamethoxazole; STR, streptomycin. **Highly statistically significant (*p* = 0.0015).

**FIGURE 2:**
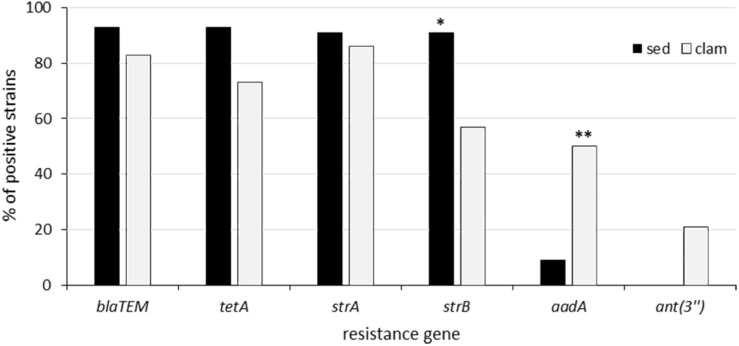
Prevalence of resistance genes among resistant clam and sediment strains. **Statistically significant (*p* = 0.03). *Not quite statistically significant (*p* = 0.09).

The results of antimicrobial susceptibility testing showed that resistant isolates were significantly (*p* < 0.05) more numerous in phylogroup A (75.6%) than in the other phylogroups (56%). The prevalence of clam and sediment strains resistant to the different antimicrobials is reported in Figure 1. Among the 53 clam strains, resistance to tetracycline was the most frequent (62%; *n* = 33), followed by resistance to ampicillin (43%; *n* = 23) and streptomycin (26%; *n* = 14) (Figure 1), in line with a previous report (Vignaroli et al., 2016). Resistance to tetracycline was significantly more frequent (*p* = 0.0015) among clam than among sediment strains (Figure 1). Multidrug resistance was detected in 15 strains (28%), most of which belonged to phylogroup A (47%), B2 (20%) or E (13%) and was not significantly associated with any phylogroup. The percentage of resistant strains positive to the relevant resistance genes is shown in Figure 2. Of the 23 ampicillin-resistant strains, 21 (91%) were ESBL producers and 19 of them (83%) carried a gene (*bla*_*TEM*_) encoding a TEM-type β-lactamase, whereas 24 out of 33 (73%) tetracycline-resistant strains were positive for *tet*(A). In the streptomycin-resistant strains (*n* = 14) the genes *strA* and *strB* were recovered at a frequency of 86% (*n* = 12) and 57% (*n* = 8), respectively, whereas the *ant*(3″) gene was found in 21% (*n* = 3) of clam strains and *aadA* (50%; *n* = 7) was significantly (*p* = 0.03) more common in clam than in sediment strains (Figure 2).

**FIGURE 3:**
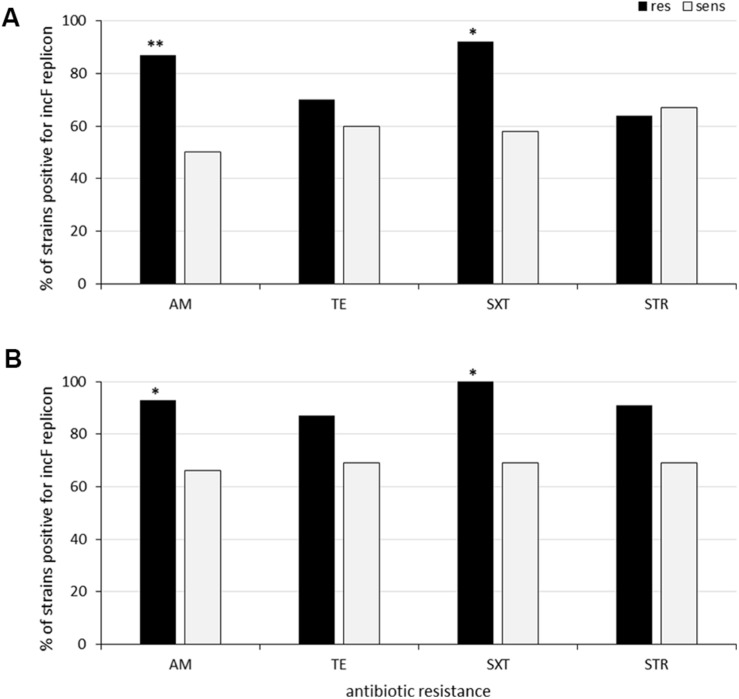
Association between antibiotic resistance and IncF family replicons among clam **(A)** and sediment isolates **(B)**. **Statistically significant (*p* = 0.007). *Not quite statistically significant (*p* = 0.07–0.09).

Altogether, 30% (*n* = 15) of sediment strains were resistant to tetracycline, 30% (*n* = 15) to ampicillin and 22% (*n* = 11) to streptomycin, in line with the results from the clam strains, which come from the same areas (Figure 1). The association between the occurrence of antimicrobial resistance and specific Inc family plasmids was then investigated. The only detected association was between ampicillin resistance and the IncF family in the clam isolates (*p* = 0.007), as shown in Figure 3.

Multidrug resistance (to three or more antibiotic classes) was also detected in 11 sediment strains (22%); most of them (82%) belonged to phylogroup B1 (36%), B2 (28%) or A (18%); however, as in the clam strains, multidrug resistance was associated with none of the phylogroups. All but one of the ampicillin-resistant strains (93%; *n* = 14) were positive to the ESBL production test and carried *bla*_*TEM*_ gene. Two strains from the PN site were also positive for a gene encoding a SHV-type β-lactamase (*bla*_*SHV*_) and an ESBL-encoding gene (*bla*_*CTX–M*_), respectively. ESBL producers were recovered from all three sites, as displayed by the phenotypic screening. Moreover, 93% (*n* = 14) of tetracycline-resistant strains carried the *tet*(A) gene and all streptomycin-resistant isolates were positive for *strA* and *strB* at a high frequency (91%; *n* = 10), like the streptomycin-resistant clam strains (Figure 2). The *ant*(3″) gene was never detected, whereas a single strain (*E. coli* PN56) carried both *aadA* and *dfrA1*. All MDR sediment strains (*n* = 11) carried multiple IncF replicons, with the FII-FIB combination being predominant (64%). All MDR clam strains but one (*n* = 14) were also IncF-positive. Most of them (*n* = 6) carried the FII replicon alone, whereas three strains carried FII and FIB.

Although 80% (*n* = 21) of MDR strains from both clam and sediment were positive for the integrase gene *intI1*, amplification of the variable region of the class 1 integron was obtained only from 20 and 9% of the clam and sediment strains, respectively. Sequence analysis of gene cassettes demonstrated three different arrangements: *dfrA1-aadA1* and *dfrA17-aadA5* in clam strains and *dfrA12-aadA2* in sediment strains.

The 14 *E. coli* isolates from clams (*n* = 9) and sediments (*n* = 5) showing positivity for the IncI1α group were subjected to S1-PFGE. Analysis of the number and size of their plasmids (Table 2) indicated that all isolates harbored 1–3 plasmids, ranging in size from 75 to 145 kb. To assess the involvement of the IncIα group in the transfer of antibiotic resistance, six tetracycline-resistant isolates carrying the *tet*(A) gene were used as donors in conjugation experiments. *E. coli* ISZ 220 from clams and *E. coli* PN30 from sediments transferred tetracycline resistance to *E. coli* 1816 at the highest frequency (respectively, 4.8 × 10^–3^ and 1.2 × 10^–7^). Five transconjugants obtained from the two mating pairs were analyzed for their plasmid profile and resistance gene acquisition. In both mating assays the transconjugants acquired all the resistance determinants and the *intI1* gene, but not all the plasmids (Table 2 and Supplementary Figure S1). In fact, S1-PFGE showed that *E. coli* ISZ 220 transferred two (∼110 and 55 kb) of its three plasmids; PBRT confirmed that the transconjugants were positive for replicons I1α and M, but not for replicon F. In contrast, *E. coli* PN30 transferred only the IncF plasmid, although it also carried an IncI1 plasmid, as shown by S1-PFGE and by PCR amplification of the replicons. The hybridization assays confirmed the location of the *tet*(A) gene on the larger plasmids of the two donors (on the 110 kb plasmid in *E. coli* ISZ 220 and on the 145 kb plasmid in *E. coli* PN30) and of the relevant transconjugants (Supplementary Figure S1).

**TABLE 2:** *E. coli* isolates from clams and sediments showing positivity for the IncI1α group: phylogroups, resistance genes and plasmid sizes.

*E. coli* strain	Phylo-group	Resistance genes	PBRT replicon	Plasmid size (∼kb)
**Clams**				
ISZ 220	C	*bla*_*TEM*_, *tet*(A), *dfrA1*, *aadA*	I1α – FIB – FII – M	110, 95, 55
ISZ 276	D	*strA*, *strB*	I1α	90
ISZ 61	E	*bla*_*TEM*_, *dfrA1*, *strA*, *aadA, ant*(3″)	I1α – FIB – FII – N	140, 80, 40
ISZ 80	F	–	I1α	85
ISZ 210	A	–	I1α – FIB	110, 95
ISZ 211	A	–	I1α – FIB – FIA	80, 40
ISZ 255	B1	–	I1α	80
ISZ 274	A	*bla*_*TEM*_	I1α	90
ISZ 325	B2	*tet*(A)	I1α	90
**Sediments**				
PN41	A	*tet*(A)	I1α	95
PN44	A	*tet*(A)	I1α	95
PN16	B1	–	I1α	85
PN29	B1	*bla*_*TEM*_, *tet*(A), *strA*, *strB*	I1α – FIB – FII	145, 75
PN30	B1	*bla*_*TEM*_, *tet*(A), *strA*, *strB*	I1α – FIB – FII	145, 75

## Discussion

The rapid evolution and global diffusion of MDR *Enterobacteriaceae* (mostly *E. coli* and *Klebsiella pneumoniae*) is raising widespread concern. Specific successful bacterial clones such as *E. coli* ST131 and *K. pneumoniae* ST258 are major causes of hospital- and community-acquired infections (Mathers et al., 2015). For epidemiological purposes, epidemic clones are usually identified by their genetic background (e.g., ST determination), whereas plasmid DNA is ignored despite its important role in strain resistance traits. The association between high-risk clones and specific resistant plasmids involved in resistance gene dissemination has been demonstrated in clinical settings (Mathers et al., 2015; Roer et al., 2018). In contrast, environmental *E. coli* isolates have not been thoroughly investigated for the diversity and transferability of antibiotic resistance plasmids, despite the fact that assessment of their plasmid profile could help identify dangerous strains and their origin. Moreover, recurrent detection of some plasmid types could indicate their role in strain survival in specific habitats as well as in the spread of antibiotic resistance traits. In this study, 103 AR strains from clam and marine sediment samples, collected along the mid-Adriatic coast, were analyzed for their plasmid content and for the transferability of plasmid-associated resistance traits. The PBRT kit employed in the study proved useful to identify the most common plasmids. Even though 14.5% of strains were negative for all the replicons targeted by the kit, most (93%) were not typable because they did not contain plasmid DNA. IncF replicons were the most frequent in our isolates, in line with reports that IncF plasmids are the most common plasmids in *Enterobacteriaceae*, especially in *E. coli* species (Carattoli, 2009). In Europe IncF plasmids are described predominantly in human and animal isolates, not in environmental strains (Rozwandowicz et al., 2018). Consequently, their high prevalence in our clam and sediment isolates may reflect their human or animal origin.

IncF plasmids are typically multireplicon, often encoding FII together with FIA and/or FIB (Villa et al., 2010). The plasmids containing FII and FIA replicon types have been described in the epidemic *E. coli* strain ST131 and in *K. pneumoniae* ST258 clones (Mathers et al., 2015). In our *E. coli* strains, most IncF-positive isolates, particularly among sediment isolates, contained the FII-FIB combination or FII alone. In a recent study (Lambrecht et al., 2018), the FII-FIB combination was found to be predominant in commensal MDR *E. coli* from farm animals, mainly broilers. IncF plasmids are often associated with IncI plasmids, which are also common in *E. coli* and *Salmonella enterica* from poultry (Rozwandowicz et al., 2018). Moreover, IncF and IncI plasmids have been reported in association with MDR *E. coli* strains, mainly ESBL producers, from food animals (Xie et al., 2016). The prevalence of both IncF and IncI plasmids in our isolates, particularly from clams, strengthen the hypothesis that contamination of our clam harvesting areas came from animal sources.

Resistance to multiple antimicrobial classes is common in *E. coli* (ECDC, 2019) and the prevalence of resistance to β-lactams, tetracycline and aminoglycosides in our clam and sediment strains is in line with earlier reports (Vignaroli et al., 2016; Pormohammad et al., 2019). Notably, antibiotic resistances are frequently associated with conjugative IncF or IncI plasmids (Rozwandowicz et al., 2018). Accordingly, in our study these Inc groups were detected at higher frequency in resistant than in susceptible strains; moreover, sediment strains exhibited a significant association between β-lactam resistance and the presence of IncF plasmids. This Inc group was also associated, besides the IncI group, with the conjugative transfer of tetracycline resistance from *E. coli* donors of both origin (clams and sediment). These plasmids were probably involved in multidrug resistance, since mating experiments resulted in the transfer of multiple resistance genes. Most MDR strains (∼80%) were positive for the class I integron and the co-transfer of the *intI1* gene and of the resistance genes suggest the plasmid location of the integron. In particular, donor *E. coli* ISZ 220 (from clams) carried an integron with the *dfrA1-aadA1* cassette that was probably linked to the ∼110 kb IncI plasmid transferred in mating assays. Plasmids containing this cassette array have been described more frequently in MDR *Salmonella* and MDR *E. coli* isolates from meat and food animals than in human isolates (van Essen-Zandbergen et al., 2009; Sunde et al., 2015). In contrast, the *dfrA17-aadA5* cassette, which was found in a single clam strain, has typically been reported in isolates of human origin (Povilonis et al., 2010; Musumeci et al., 2012; Sunde et al., 2015). This finding may also be ascribed to contamination of the sampling areas with fecal bacteria mostly of animal origin. Therefore, the presence of a class I integron on conjugative plasmids contributes both to the emergence of MDR strains and to the dissemination of antibiotic resistance.

## Conclusion

In conclusion, to the best of our knowledge this is one of the few studies focused on the prevalence of specific Inc group plasmids in *E. coli* isolates from secondary habitats (like clams and sediments). The PBRT kit, which has been developed for human isolates *of Enterobacteriaceae*, proved a useful tool to type the plasmids conferring antibiotic resistance on environmental *E. coli* isolates, to predict their origin and to formulate hypotheses on the contamination source. Moreover, these data could help correlate a plasmid type to strain adaptation and survival strategies outside the host, and provide further information on the spread of antibiotic-resistant plasmid families among *Enterobacteriaceae* in different settings.

## Data Availability Statement

The raw data supporting the conclusions of this article will be made available by the authors, without undue reservation, to any qualified researcher.

## Author Contributions

CV, BC, FA, and FB designed the experiments, analyzed the results, and drafted the manuscript. MM contributed to result interpretations. SS, EC, GM, and NC performed the sampling and the experiments.

## Conflict of Interest

The authors declare that the research was conducted in the absence of any commercial or financial relationships that could be construed as a potential conflict of interest.

## Footnotes

1 http://blast.ncbi.nlm.nih.gov/Blast.cgi

2 https://pubmlst.org/plasmid
