# Supplementary material for: Plasmid Replicon Typing of Antibiotic-Resistant Escherichia coli From Clams and Marine Sediments
Source: Front Microbiol. 2020 May 27;11:1101. doi: 10.3389/fmicb.2020.01101 (PMC7266932; doi:10.3389/fmicb.2020.01101)
Supplement: Supplementary file 1 [file Image_1.pdf]

Figure S1

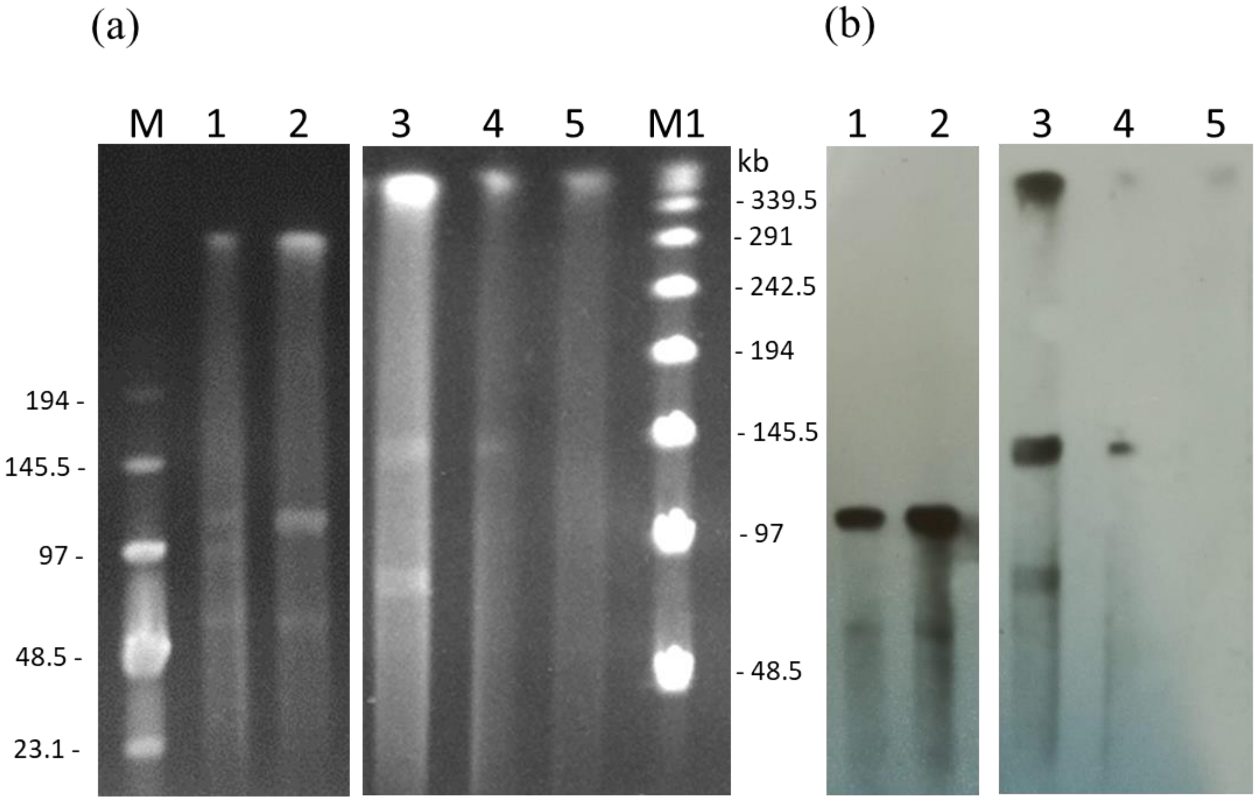

S1-PFGE plasmid profiles of donors, transconjugants and the recipient (a), and hybridization with the *tet*(A) probe (b).

M, DNA molecular weight Low Range PFG marker; M1, Lambda Ladder PFG Marker (New England Biolabs, Ipswich, MA, USA).
Fragment size (kb) is reported on either sides of the figure (a); lane 1, donor *E. coli* ISZ 220; lane 2, transconjugant *E. coli* 220x1816(5); lane 3, donor *E. coli* PN30; lane 4, transconjugant *E. coli* 30x1816(3); lane 5, recipient *E. coli* 1816.
